# Fast chromium determination in pharmaceutical tablets by using electrochemical sensors: Preparation and comparison

**DOI:** 10.1016/j.heliyon.2023.e22842

**Published:** 2023-11-24

**Authors:** Abbas Nasri Fateh, Leila Hajiaghababaei, Mohammad Reza Allahgholi Ghasri, Ashraf Sadat Shahvelayati, Khadijeh Kalateh

**Affiliations:** Department of Chemistry, Yadegar-e-Imam Khomeini (RAH) Shahre Rey Branch, Islamic Azad University, Tehran, Iran

**Keywords:** Chromium, Coated wire electrode, Liquid membrane electrode, N-(pyridine-2-ylcarbamothioyl) benzamide, Potentiometry sensor, Solid-state electrode

## Abstract

In the present paper, three electrodes were prepared with the aim of detecting chromium (III) in pharmaceutical tablets and comparing their capabilities and efficiency. At first, N-(pyridine-2-ylcarbamothioyl) benzamide (NP2YCTB) was synthesized and characterized by ^1^H NMR, FTIR, and ^13^C NMR spectroscopy methods. Then, it is used as a sensing material to prepare three types of chromium potentiometry sensors including solid-state electrodes (SSE), coated wire electrodes (CWE) as asymmetric electrodes, and liquid membrane electrodes (LME) as symmetric electrodes. The responses of all electrodes were Nernstian. Field-emission scanning electron microscopy was utilized to investigate the liquid membrane morphology. The presence of chromium (III) in the membrane was proved using Energy-dispersive X-ray spectroscopy and the coordination of NP2YCTB heteroatoms with chromium (III) was confirmed by Fourier transform infrared spectroscopy. The limit of detection for SSE (3 × 10^−9^ mol/L) was enhanced compared with LME (7 × 10^−6^ mol/L) and CWE (3 × 10^−7^ mol/L). The response time of electrodes was very short so it was about 5–6 s for LME and CWE and 5–8 s for SSE. The sensors were used for the potentiometric determination of chromium (III) in pharmaceutical tablets and in the potentiometric titration of it with EDTA.

## 1 Introduction

Chromium (III) is a key ion for industrial samples and biological systems. Chromium (III) compounds have a main role in the metabolism of certain lipids and glucose, mostly cholesterol [1,2]. Chromium (III) ion is a key nutrient for humans. However, health effects can be caused by the uptake of a huge deal of chromium (III). According to several in vitro studies, DNA damage can be caused by the higher concentrations of chromium (III) in the cell [3]. Generally, sophisticated analytical approaches such as atomic absorption spectroscopy (AAS), inductively coupled plasma-atomic emission spectroscopy (ICP-AES), and X-ray fluorescence (XRF) [[4], [5], [6]] are used for determining the trace level of metals like chromium. However, these methods have some disadvantages including the time-consuming and tedious procedure of sample preparation and the cost of routine analysis. Thus, it is a challenging task to search for new, simple, and fast approaches to the measurement of chromium [7].

The potentiometric sensors have become appropriate tools in the analysis of different species owing to the simplicity and speed of working [[8], [9], [10]]. Asymmetric and symmetric sensors can be made using the technique to immobilize the polymeric membrane on the electrode. Generally, common PVC membrane electrodes require an internal filling solution and an internal reference electrode. Due to the placement of the membrane between two solutions, the possibility of the leaching of the membrane ingredients to the aqueous solutions on its two sides increases. Moreover, the electrode had low mechanical stability. However, by coating the polymeric membrane on a conducting wire's surface [11,12], the internal solution and internal reference electrode are not required. By removing the internal solution, the leaching process is reduced thus reducing the low detection limit [13]. In the coated wire electrodes, a thin polymeric film containing an ionophore is dip-coated directly on the metal wire [14]. Such electrodes represent the primary creation of the solid-state ion-selective electrode. Though the coated wire electrode is prepared simply, the potentials drift chaotically due to the sensitivity to small accidental charges as well as the “blocking” interfaces of membrane/wire [15]. Since water may permeate through the membrane, there is a possibility of a thin water layer forming on the surface of the wire. The electrode potentials' instability is a result of the unstable metal ion concentration in the aqueous layer. Hence, using an electron-ion exchanger has been highly focused on enhancing the electrode performance and “blocking” interfaces [16]. Owing to their good conductivity and hydrophobicity, carbon-based nanomaterials are used extensively as electron-ion exchangers. Drift and the creation of water films were exhibited by potentiometric sensors oriented by a multi-walled carbon nanotube (MWCNT) as the inner transducing layer [17,18].

The ionophore used is very important in designing a potentiometric sensor. Nano-chitosan, rhodamine-B chromate, and 4-(5-bromothiophen-2-carboxylidene amino)-3-methyl-5-mercapto-*s*-triazole [[19], [20], [21]] are examples of ionophores used to prepare chromium (III) electrodes. However, a large section of these potentiometric electrodes has some defects such as high detection limit, narrow working pH range, long response time, short life span, and serious interference from other cations.

**Fig. 1:**
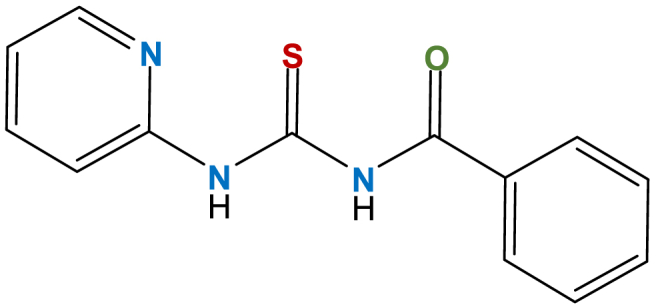
N-(pyridine-2-ylcarbamothioyl) benzamide (NP2YCTB).

Therefore, we aimed to synthesize N-(pyridine-2-ylcarbamothioyl) benzamide (NP2YCTB) (Fig. 1) and utilize it as a novel sensing material. NP2YCTB forms a good complex with chromium (III), which makes it a suitable and selective sensing material for use in the liquid membrane of chromium (III)-selective electrode. Hence, we prepared three various liquid membrane chromium (III)-selective electrode designs including solid-state electrode (SSE) with conductive polymer composite containing multi-walled carbon nanotube, coated wire electrode (CWE), and classic liquid membrane electrode (LME) with liquid internal electrolyte. Various selectivity behaviors and compositions were investigated. The performance and ability of the symmetric and asymmetric electrodes were compared to measure chromium (III) concentration. Using Field-emission scanning electron microscopy-Energy-dispersive X-ray spectroscopy (SEM-EDX), the PVC liquid membrane's morphology was investigated. Fourier transform infrared (FTIR) spectra were utilized for studying coordinated sites of NP2YCTB to chromium (III).

To the best of our knowledge, these three novel membrane electrodes are the first electrodes created with NP2YCTB as the electrode's recognition component.

## 2 Experimental

### 2.1 Materials and apparatus

Section S1 describes all apparatus and materials.

### 2.2 Synthesis of the NP2YCTB

Benzoyl chloride and potassium thiocyanate were dissolved in acetonitrile and it was stirred at room temperature for 30 min. After separation and filtration, the solid residue was then utilized without purification. Benzoyl isothiocyanate (2 mmol) and 2-aminopyridine (2 mmol) in 10 mL acetonitrile were mixed and refluxed, for 8 h at 60 °C. The product formation was confirmed using TLC. The solvent was evaporated after completing the reaction, under decreased pressure. Crystallizing from n-hexane/ethyl acetate led to the purification of the yellowish crude product (NP2YCTB) (Fig. 1), which was utilized in the composite of the membrane. It was followed by the structural confirmation by measuring the melting point, ^1^H NMR, FTIR, and ^13^C NMR spectroscopy methods.

NP2YCTB: Yellow crystals, m.p. 142–144 °C; yield: 96 %. IR (KBr): 3350, 3220, 1696, 1673, 1580, 1467, 1266, 1189, 882, 760 cm-1. ^1^H NMR (300 MHz, CDCl3): δ = 7.15 (1 H, dd, 3J = 5.2, 3J = 6.8, CHPy), 7.53 (2 H, t, 3J = 7.3, 2 CH), 7.61 (1 H, d, 3J = 5.2, CHPy), 7.75 (1 H, t, 3J = 7.3, CH), 7.87 (2 H, d, 3J = 7.3, 2 CH), 8.43 (1 H, d, 3J = 5.2, CHPy), 8.80 (1 H, d, 3J = 8.2, CHPy), 9.14 (1 H, s, NH), 13.10 (1 H, s, NH) ppm. ^13^C NMR (300 MHz, CDCl_3_): δ 116.0 (CH), 121.4 (CH), 127.5 (2 CH), 129.1 (2 CH), 131.6 (C), 133.7 (CH), 137.7 (CH), 148.5 (CH), 151.2 (C), 166.4 (C=O), 177.0 (C=S) ppm. Anal.Calcd for C13H11N3OS (257.3): C, 60.68; H, 4.31; N, 16.33; O, 6.22; S, 12.46. Found: C, 61.22; H, 4.95; N, 16.72; S, 12.89.

### 2.3 Investigating the ionophore selectivity by UV–Visible spectrophotometry

UV–visible spectrophotometry was utilized to assess the NP2YCTB interaction with some metal cations. The spectrum of the 5 × 10^−5^ mol/L of NP2YCTB in acetonitrile was recorded. Then, 300 μL aliquots of different cations solutions (1 mmol/L) were added to this solution and the spectral changes in the NP2YCTB spectrum were defined. To record the spectrum of chromium (III) alone, 300 μL aliquots of chromium (III) (1 mmol/L) were added to acetonitrile, and the spectrum was recorded.

### 2.4 Preparation of the ion-selective electrodes

The NP2YCTB, PVC, ionic additive, and plasticizer were dissolved in THF with the ratio presented in Table 1 for the fabrication LME. Then, mixing the solution was performed in a small beaker. THF was then evaporated gradually to achieve a viscous solution. For about 5 s, some plastic tubes with a diameter of 3–5 mm were separately immersed in the mixtures to create a transparent membrane about 0.3 mm thick. Then, the plastic tube was put for about 12 h at room temperature. Then, the specimens were filled with 0.01 mol/L Cr(NO_3_)_3_ solution as internal filling. Finally, by soaking in a 1.0 mmol/L Cr(NO_3_)_3_ solution, the electrodes were conditioned for 24 h.

To make a CWE, a copper wire with 10 cm length and 0.5 mm diameter was polished and immersed 3 times in a viscous solution obtained from the composition of 2%NaTPB, 10 % NP2YCTB, 58 % DBP, and 30 % PVC. It was then left to dry for 12 h in the air. By soaking in a 1.0 mmol/L Cr(NO_3_)_3_ solution, the prepared membrane electrode was conditioned for 24 h.

There were three parts in making the SSE including section A: copper wire (unshielded and with 10 cm length and 0.5 mm diameter); section B: conductive composite as an internal and transducer contact; and section C: PVC liquid membrane. For constructing a conductive composite, the carbon nanotube (3 %), hardener (15 %), powdered graphite (47 %), and epoxy resin (35 %) were mixed in THF. The mixture was left in the air for 30 min. A mixture of hardener and epoxy resin was used to bind the graphite. Copper wire was dipped into this mixture 10 times. The wire was coated with the mixture and left to dry for 12 h. The performance of the transducer is improved using a carbon nanotube in this composition. This material was then immersed 3 times into the liquid membrane cocktail (section C) which was made with 30 % PVC, 10 % NP2YCTB, 58 % DBP, and 2%NaTPB. Next, it was air-dried for 12 h. Ultimately, the electrode was submerged in 1.0 mmol/L Cr(NO_3_)_3_ solution for 24 h. In subsequent measurements, conditioning was followed by a second step in a chromium (III) solution of 0.01 mmol/L.

### 2.5 Analytical process of real specimens

To examine the symmetric and asymmetric electrodes’ applicability to real samples, chromium (III) amounts were determined by the electrodes in pharmaceutical tablets. Tablets contained 500 μg and 200 μg of chromium. Five of each tablet was separately powdered. Each sample was solved in distilled water (500 mL). The samples were stirred for 5 min and slightly heated. Then they were filtered and their pH was adjusted to 5 by adding a few drops of hydrochloric acid. The samples were analyzed using a potentiometric technique using the electrodes and flame atomic absorption spectroscopy (FAAS).

## 3 Results and discussion

In this work, the best polymeric membrane composition as a sensing layer was first determined. Then the effect of different transducers on the chemical-to-electrical signal transmission of a potentiometric membrane electrode was investigated. Of course, before these things, using UV–Vis spectrophotometry, the affinity of the sensing element (NP2YCTB) to some cations was investigated.

### 3.1 UV–visible spectrophotometry

**Fig. 2:**
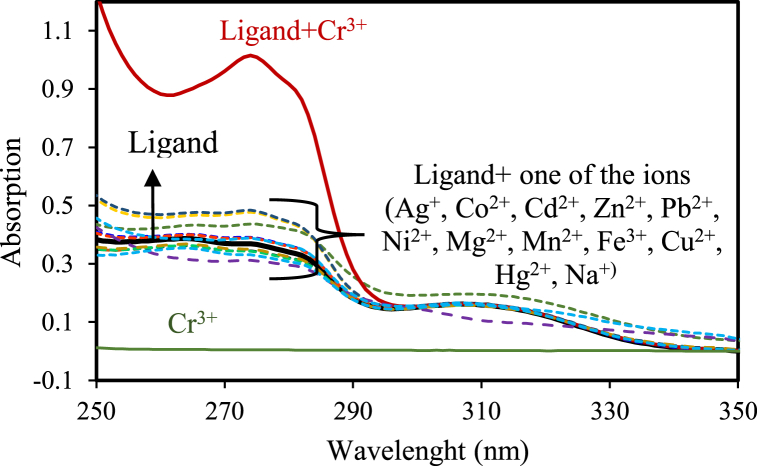
The changes in UV–Vis spectra of NP2YCTB solution (5 × 10^−5^ mol/L) followed by adding 300 μL of various cations solutions (1 mmol/L).

NP2YCTB contains one oxygen, three nitrogen, and one sulfur atom in its structure. Therefore, the probability of its complexation with transition or soft metals is relatively high. UV–Vis spectrophotometry was used for scrutinizing the NP2YCTB affinity to various cations before starting the construction of the PVC membrane electrode [22,23]. Hence, equivalent amounts of various ions were inserted separately into NP2YCTB solutions. Recording the UV–Vis spectra (Fig. 2) the changes in absorption were assessed. Hence, the change in absorption intensity of NP2YCTB after the addition of chromium (III) is much greater than when other cations are added, this can be a confirmation of higher affinity between chromium (III) and NP2YCTB compared to other cations. Therefore, NP2YCTB can be considered as a good sensing element or ionophore for fabricating PVC membrane chromium (III)-selective electrodes.

### 3.2 Sensing element composition

For evaluation of the composition, several LMEs based on NP2YCTB with different compositions of the membrane were prepared and their response features were assessed (Table 1).

**Table 1:** Optimizing the membrane ingredients.

NO.	% Plasticizer	% NP2YCTB	% PVC	% NaTPB	Slope, mV/decade
1	DBP (70)	0	30	0	2.1 ± 0.3^a^
2	DBP (68)	0	30	2	4.7 ± 0.5
3	DBP (60)	5	33	2	12.9 ± 0.5
4	DBP (58)	10	30	2	20.7 ± 0.6
5	DBP (56)	12	30	2	14.9 ± 0.7
6	DBP (55)	13	30	2	12.6 ± 0.6
7	DBP (54)	13	30	3	15.8 ± 0.5
8	DBP (58)	10	32	0	16.9 ± 0.8
9	NB (60)	10	30	2	8.3 ± 0.4
10	NB (56)	12	30	2	9.5 ± 0.4
11	NB (54)	13	30	3	10.4 ± 0.5

a SD is based on three replicate measurements.

The presence of high concentrations of the ionophore causes the extraction of chromium (III) ions into the liquid membrane. As seen in Table 1, when there is no NP2YCTB (no. 1 and 2) in the membrane, the response can be neglected. By increasing the amount of it in the membrane up to 10 % wt., the slope of the electrode increases (no. 4), and this shows the affinity of NP2YCTB to chromium (III). However, the sensor's response (no. 5–7) is reduced by adding ionophore more due to the possible saturation of the membrane and inhomogeneity [22].

The response features of ion-selective electrodes were highly reported influenced by the amount and nature of the used plasticizer. The reason is the effects of plasticizers on the membrane phase's dielectric constant and the mobility and state of the ionophore molecules [24]. The membrane composition is homogenized by the plasticizer, facilitating the ionophore's diffusional mobility within the membrane. Two plasticizers of NB with a high dielectric constant (DC = 34.8) and DBP with a low dielectric constant (DC = 6.4) were utilized. According to Table 1, DBP had better performance than NB (no. 4 and 9). It seems that the high affinity of NP2YCTB to chromium (III), which is an ion with a high charge density, compensates for its poorer extraction by the DBP-containing membrane (with a lower dielectric constant) compared to the NB-containing membrane (with a higher dielectric constant). Furthermore, the polar interfering ions' lower extraction which can compete with the chromium (III) in complexation with NP2YCTB, was resultant from the DBP's lower dielectric constant. It could have positive effects on the selectivity behavior of the sensor.

It was indicated that the presence of lipophilic additives with negative charge can increase the potentiometric performance of cation-selective electrodes by reducing the ohmic resistance. Besides, additives may catalyze the exchange kinetics at the interface of the sample and membrane. Also, additives can enhance the poor extraction capability of some ionophores. As seen in Table 1, adding 2 % NaTPB caused a Nernstian performance of the electrode (no. 4 and 8).

As seen in Table 1, the Nernstian slope of 20.7 mV/decade was yielded by membrane number 4 with NP2YCTB: DBP: PVC: NaTPB percent ratio of 10:58:30:2. Ultimately, CWE and SSE were fabricated using this optimal membrane composition.

### 3.3 pH effect on chromium (III)- symmetric and asymmetric electrodes

**Fig. 3:**
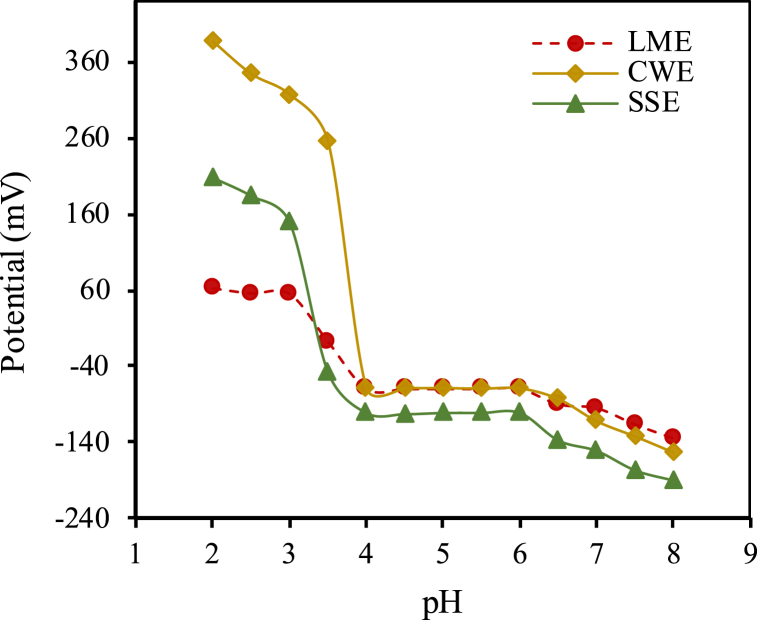
The effects of pH on the response of three kinds of electrodes.

pH effect on the response of three kinds of electrodes in a solution containing 0.1 mmol/L chromium (III) was investigated in the range of pH 2.0 to 8.0. To set the pH, a concentrated solution of hydrochloric acid or sodium hydroxide was used. According to the findings (Fig. 3), the range of pH 4 to 6.0 is the working pH range of all three electrodes because the potential remains constant in this range. The potentials were reduced owing to the non-completing complexation reaction or hydrolysis of chromium (III) at higher and lower than this working pH range. This trend same was the same in all three kinds of sensors. In this study, solutions of chromium with known concentrations were prepared in distilled water and used to optimize the parameters affecting potentiometric measurement and to draw the calibration curve. Also, some chromium solutions were prepared by dissolving pharmaceutical tablets in distilled water. Before potentiometric measurement, the pH of all prepared solutions was adjusted to 5 by adding a concentrated solution of hydrochloric acid or sodium hydroxide to them.

### 3.4 Calibration curves

**Fig. 4:**
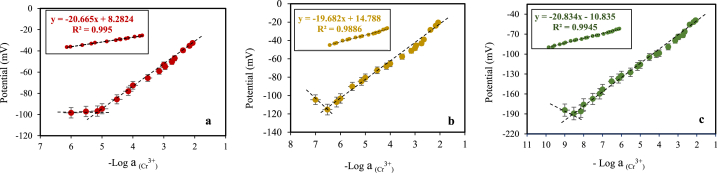
The calibration curves of a) LME, b) CWE, and c) SSE.

The best membrane's composition was immobilized on the unshielded copper wire (to prepare coated wire electrode) and conductive composite (to prepare solid-state electrode) to improve the linear concentration range of the LME. As seen in Fig. 4a, b and c, the LME showed a slope of 20.7 mV/decade in a concentration range of 1 × 10^−1^ mol/L to 7 × 10^−6^ mol/L of chromium (III) (Fig. 4a). The CWE showed a slope of 19.7 mV/decade in a concentration range of 1 × 10^−1^ mol/L to 3 × 10^−7^ mol/L of chromium (III) (Fig. 4b) and SSE showed a slope of 20.8 mV/decade in a concentration range of 1 × 10^−1^ mol/L to 3 × 10^−9^ mol/L of chromium (III) (Fig. 4c). Considering that the optimal value of the Nernstian slope is 59.10/n (mV/decade), where (n) shows the valency [19], all three electrodes show the Nernstian slope for chromium (III) determination. However asymmetric electrodes (CWE and SSE) can measure a wider concentration range than symmetric electrodes (LME).

The detection limits were calculated by substitution of the potential value, which is the cut-off point projection in the correct equation. A low detection limit was displayed by the sensors including 7 × 10^−6^ mol/L, 3 × 10^−7^ mol/L, and 3 × 10^−9^ mol/L for LME, CWE, and SSE, respectively. The results show that these asymmetric electrodes, in addition to having higher mechanical stability, can be used to measure a wider concentration range compared to symmetric electrodes (LME). Also, using them decreases the detection limit of the method. In the symmetric electrode, because the membrane is in contact with the chromium (III) solution from both sides, transmembrane ion fluxes from the inner filling solution into the sample solution are higher than in the asymmetric electrode which has no inner solution. This limits the detection limit of the symmetric electrode [25]. On the other hand, CNT in SSE design increases surface area and also allows for improved mass transport of the target analyte to the electrode surface. The enhanced mass transport can lead to a higher sensitivity and lower detection limit as more analyte molecules can reach the electrode. Statistics of the response characteristics of the CWE, LME, and SSE to chromium (III) are summarized in Table 2.

**Table 2:** Statistics of the response characteristics of the CWE, LME, and SSE to chromium (III).

Parameters	LME	CWE	SSE
Calibration line equation	Y = −20.665X+8.2824	Y = −19.682X+14.788	Y = −20.834X+10.835
Slope (mV/decade)	20.7	19.7	20.8
Correlation coefficient, R^2^	0.995	0.9886	0.9945
Detect Limit (M)	7 × 10^−6^	3 × 10^−7^	3 × 10^−9^
Working linear Range (M)	7.0 × 10^−6^–1.0 × 10^−1^	3.0 × 10^−7^– 1.0 × 10^−1^	3.0 × 10^−9^– 1.0 × 10^−1^

### 3.5 Response time and lifetime of chromium (III)-electrodes

**Fig. 5:**
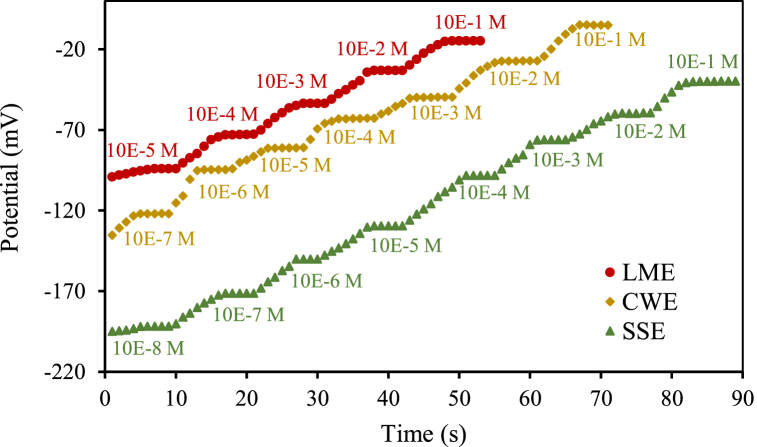
The response time of proposed LME, WCE, and SSE.

Electrode response time in a potentiometric method is defined as the time required for the potential response to reach values within ±1 mV of the ultimate equilibrium potential [13]. The potential changes of the chromium (III) electrodes are recorded to obtain this value by succeeding immersion in some chromium (III) solutions, each with a 10-fold difference in concentration. All three electrodes could reach quickly their equilibrium response in the whole concentration range. Response time for LME and CWE is about 5–6 s and for SSE is about 5–8 s (Fig. 5).

The stability and lifetime of the symmetric and asymmetric electrodes were investigated. From each type, three similar electrodes were selected and their slopes were determined during 14 weeks. Electrodes worked about 1 h a day during this time. It was indicated that the LME can be used for at least 10 weeks with no significant alterations in its slope, which was 12 and 11 weeks for SSE and CWE. After this time, the Nernstian slope was decreased. The increase in the lifetime of asymmetric electrodes compared to the symmetric electrode can be due to the reduction of the leaching procedure due to the elimination of the internal solution.

### 3.6 Interference and selectivity

The response of the ion-selective electrode to a specific ion is one of its key features and is determined over other ions and species existing in the solution. It is expressed as a selectivity coefficient. In this work, using the matched potential method (MPM) [26], the selectivity coefficients of the developed electrodes were evaluated (Table 3). A KMPM value of 1.0 represents the sensor revealing similar responses to both the primary and interfering ions, although smaller values represent its higher selectivity. The results are all much less than 1.0, indicating the developed electrodes’ high selectivity for chromium (III).

**Table 3:** The selectivity coefficients for CWE, LME, and SSE.

Ion	K_MPM_		
	LME	CWE	SSE
Zn^2+^	9.99 × 10^−4^	1.20 × 10^−2^	7.93 × 10^−3^
Ag^+^	3.16 × 10^−3^	3.16 × 10^−3^	1.89 × 10^−3^
Pb^2+^	9.69 × 10^−4^	8.57 × 10^−3^	4.91 × 10^−3^
Ni^2+^	2.24 × 10^−3^	7.94 × 10^−2^	7.84 × 10^−3^
Mn^2+^	1.97 × 10^−3^	1.99 × 10^−4^	1.48 × 10^−3^
Co^2+^	3.97 × 10^−3^	2.51 × 10^−3^	5.61 × 10^−3^
Na^+^	2.00 × 10^−4^	2.81 × 10^−4^	1.00 × 10^−4^
Fe^3+^	3.16 × 10^−3^	3.16 × 10^−3^	1.92 × 10^−3^
Ca^2+^	7.93 × 10^−4^	2.81 × 10^−4^	1.50 × 10^−4^
Cd^2+^	6.30 × 10^−3^	2.51 × 10^−3^	6.30 × 10^−3^
Hg^2+^	3.16 × 10^−3^	3.16 × 10^−3^	1.65 × 10^−3^
Cu^2+^	1.90 × 10^−3^	2.51 × 10^−3^	8.01 × 10^−3^

### 3.7 FESEM-EDX of liquid membrane

**Fig. 6:**
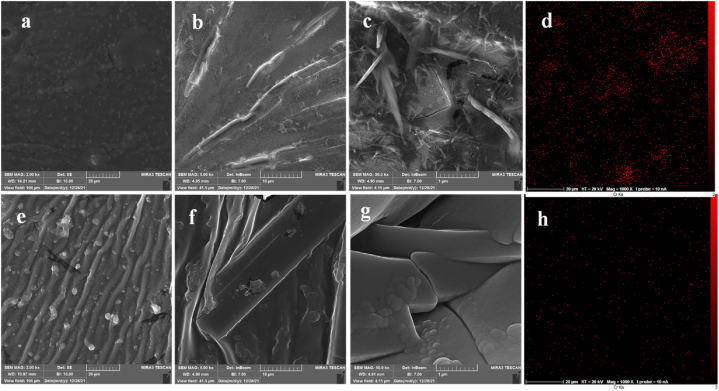
Various zooms of the FESEM micrographs of a, b, c) Liquid membrane contain NP2YCTB, and e, f, g) Liquid membrane without NP2YCTB. EDX mapping images of liquid membrane in contact with chromium (III) solution d) without NP2YCTB, h) containing NP2YCTB.

Using the FESEM images, the liquid membrane was studied. Various zooms of the FESEM micrographs of liquid membrane contain NP2YCTB and without NP2YCTB is shown in Fig. 6a, b, c, and Fig. 6e, f, and g, respectively. The membrane morphology was changed significantly followed by utilizing NP2YCTB in the liquid membrane.

The bright spots in the EDX mapping images of the liquid membrane presented in Fig. 6d and h correspond to the presence of chromium in the membrane. In the membrane comprising NP2YCTB, a significantly higher amount of chromium (III) exists compared to its amount in the membrane without NP2YCTB [27]. Therefore, the operative contribution of the NP2YCTB as sensing chromium (III) is confirmed in the liquid membrane.

**Fig. 7:**
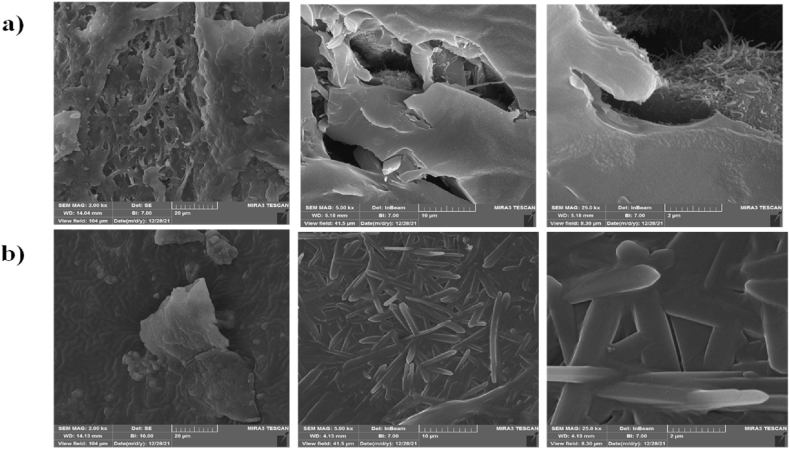
FESEM micrographs zoom of a) copper wire with just conductive polymer coating, and b) copper wire with liquid membrane containing NP2YCTB and conductive polymer coating.

Moreover, different zooms of FESEM micrographs taken of copper wire coated just with conductive polymer composite and wire coated with both conductive polymer and liquid membrane are shown in Fig. 7a and b. The carbon nanotube is revealed in the composite (Fig. 7a). Fig. 7b represents a very high coverage of liquid membrane on the coated wire.

### 3.8 Sensing mechanism interpretation

The sensing element (NP2YCTB) at the electrode surface interacts with the analyte according to the working mechanism general principles of the potentiometric ion-selective electrodes. Sensing element up-takes analyte to the electrode surface during a potentiometric experiment. Thus, the electrochemical potential of the analyte is increased on the surface of the electrode in comparison to the solution layer around the electrode. Hence, across the electrode/solution interface, an electrical potential was made. For the present membrane electrodes, the NP2YCTB plays the role of sensing element. The active sites present in NP2YCTB molecule are hetero atoms of N, O, and S able to have coordination with chromium (III).

**Fig. 8:**
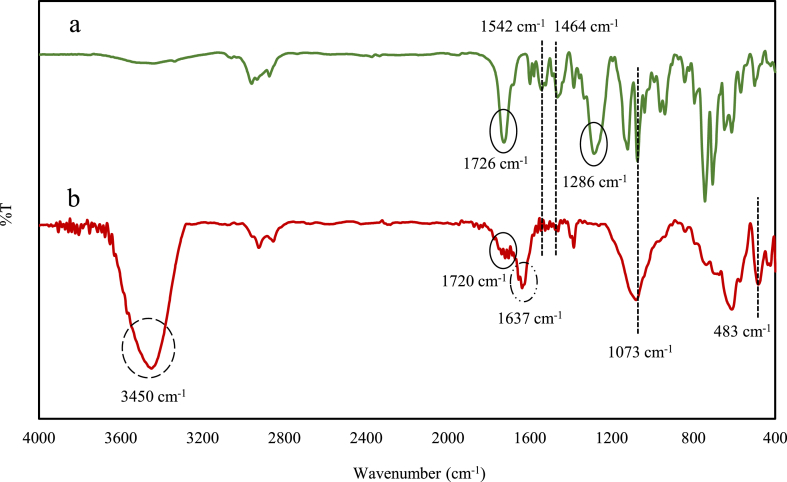
a) FTIR spectra of membrane before soaking in chromium (III) solution, b) FTIR spectra of membrane after soaking in chromium (III) solution.

Using a thin membrane layer containing the best composition, FTIR spectra were recorded before using it for sensing chromium (III) and after (Fig. 8a and b). The band around 3450 cm^-l^ is a result of bending vibration of O–H in water molecule adsorbed at the liquid membrane. But, significant changes in the FTIR spectrum of the liquid membrane contacting with chromium (III), show that there is a good interaction between NP2YCTB and chromium (III) in this membrane matrix [28,29]. The decrease in *ν*(C=O) bond strength and its redshift from 1726 to 1720 cm^−1^ confirm the coordination of the oxygen atom of the NP2YCTB molecule with chromium (III). Chromium (III) is an electropositive ion and therefore pulls the electron pair of the oxygen atom towards itself and thereby the C=O bond strength decreases [20]. Venkataraghavan and Rao assigned -N-C=S bands caused by mixed vibrations in the regions of 940–1140 cm^-l^, 1270-1420 cm^-l^, and 1395-1570 cm^-l^ in derivatives of thiocarbonyl where the C=S group is attached to one or two nitrogen atoms [30]. Therefore, the binding of chromium (III) with the thiocarbonyl group of NP2YCTB is represented by the disappearance of the peaks in 1464–1542 cm^-l^ and 1286 cm^-l^ and increasing the peaks' strength at 1073 cm^-l^. The interaction of nitrogen atoms of the NP2YCTB with chromium (III) can be caused by the arrival of new bonds at the wavelength of 483 cm^-l^ and 1637 cm^-l^ corresponding to Cr–N stretching vibration and NH out-of-plane vibration respectively in the structure of the NP2YCTB [[31], [32], [33]]. As chromium (III) ion is a hard acid in Pearson classification, it is expected to have stronger interactions with the hard oxygen atoms of NP2YCTB. The structure of the complex is probably such that chromium is closer to the oxygen atom of ionophore and has a stronger interaction with it.

### 3.9 Analytical applications

Ethylenediaminetetraacetic acid (EDTA) solution with a concentration of 1.0 × 10^−2^ mol/L was used as titrant to potentiometric titration of 20 mL of chromium (III) solution with a concentration of 1.0 × 10^−4^ mol/L. Proposed symmetric and asymmetric chromium (III) electrodes were used as indicator electrodes in these potentiometric titrations.

**Fig. 9:**
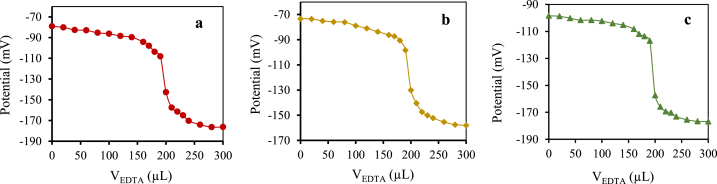
Curves of potentiometric titration of 20 mL chromium (III) solution (1.0 × 10^−4^ mol/L) with EDTA (1.0 × 10^−2^ mol/L) by using a) LME, b) CWE and c) SSE.

According to Fig. 9a, b and c, the potential values are reduced by incrementing the amount of EDTA. This decrease is due to the formation of a complex between the EDTA and chromium (III), which leads to a decrease in the concentration of chromium (III) ions in the solution. Such chromium (III) sensors could successfully have the role of indicator electrodes as a result of the sharp endpoint in the titration curve.

To further assess the possibility of using the suggested membrane electrodes in the determination of chromium (III) in real samples, the active chromium (III) ingredients were determined in some pharmaceutical tablets. As seen in Table 4, there is a good consistency between the real chromium (III) quantity in the pharmaceutical tablets and determined chromium (III) by proposed sensors.

**Table 4:** Chromium (III) determination in pharmaceutical tablets.

Sample			Chromium amount (μg/tablet)*		
	Stated Content	Found by LME/Recovery%	Found by CWE/Recovery%	Found by SSE/Recovery%	Measured by FAAS/Recovery%
Tablet 1	500	499.2 ± 0.7/99.8	499.2 ± 0.9/99.8	499.2 ± 1.1/99.8	499.4 ± 1.6/99.9
Tablet 2	200	199.0 ± 1.3/99.5	199.4 ± 1.0/99.7	200.2 ± 1.8/100.1	199.9 ± 1.9/99.9

*The results are average of three replicates measurements.

The accuracy of the chromium (III) determination was examined by taking specimens from the same batch. They determined three times via the provided SSE and by flame atomic absorption spectrometry (FAAS).

Afterwards, a student t-test was applied to compare the results. The t parameter calculated based on experimental results (t_exp_) was 0.13 in tablet 1 and 0.10 in tablet 2. Statistical parameter extracted from the t-distribution table for 4 freedom degrees and confidence level of 95 % (t = 2.78) was bigger than both t_exp_. This means that the null hypothesis is confirmed and the results obtained from the two methods are the same at the 95 % confidence level.

Also, three chromium (III) standard solutions with the same concentration were prepared and the chromium concentration in each of them was determined by an electrode three times. According to these results, relative standard deviation values were calculated and the repeatability of the proposed electrodes was estimated. The RSD values were 3.0 % by LME, 2.5 % by CWE, and 2.4 % by SSE. Reproducibility of the methods was estimated by fabricating the three electrodes from each type and analyzing a chromium (III) standard solution three times. The RSD values were 4.1 %, 3.9 %, and 3.5 % for LME, CWE, and SSE, respectively.

### 3.10 Comparison with previous studies

We compared the suggested SSE with some other chromium (III) electrodes [7,21,[34], [35], [36]] indicating the excellence of the proposed electrode. The detection limit was improved along with the working range, response time, and Nernstian slope for the proposed SSE (Table 5). It is close to most of the electrodes considering the useable pH range.

**Table 5:** Comparison between the provided SSE and some other chromium (III)-selective electrodes.

Ligand	Working range (mol/L)	DL* (mol/L)	Slope (mV decade^−1^)	pH	R.T** (s)	Ref.
4-(5-bromothiophen-2-carboxylidene amino)-3-methyl-5-mercapto-*s*-triazole	2.0 × 10^−7^ to 1.0 × 10^−1^	9.0 × 10^−8^	19.5	2–5	10–15	21
1- [(2- hydroxy ethyl) amino]-4-methyl-9H-thioxanthen-9-one	3.2 × 0^−7^ to 1.0 × 10^−1^	1.6 × 10^−7^	20.51	4.8–6.3	8	7
N, N-bis(salicylidene)-ophenylenediaminate chromium (III)	7.5 × 10^−6^ to 1.0 × 10^−2^	1.8 × 10^−6^	20.1	4.5–7.7	8	34
5,5′-(1,4-phenylene) bis (3-(naphthalen-1-yl)-4,5-dihydro-1H-pyrazole-1-carbothioamide)	1.0 × 10^−5^ to 1.0 × 10^−1^	1.7 × 10^−6^	–	5–11	8	35
N-(1-(4-bromophenyl)-3-oxo-3-phenylpropyl) acetamide	5.0 × 10^−7^ to 1.0 × 10^−3^	3.1 × 10^−7^	19.5	4.0–6.5	<15	36
N-(pyridine-2-ylcarbamothioyl) benzamide	3 × 10^−9^ to 1 × 10^−1^	3.0 × 10^−9^	20.8	4–6	5–8	This work

*Detect Limit, ** Response Time.

Also, numerous analytical techniques were compared to the suggested [6,[37], [38], [39]]. Based on the outcomes, the suggested potentiometric method's LOD is superior to flame atomic absorption spectroscopy and spectrophotometry [37,38] and is comparable to inductively coupled plasma and voltammetry [6,39]. It is noteworthy that the inductively coupled plasma and voltammetry methods require much more expensive devices than the potentiometric method. Yet it was found that the operating range and useable pH range have improved (Table 6). As a result, compared to some of the methodologies listed in Table 6, the potentiometric method with SS-ISE developed in the present work performs better without employing the preconcentration or extraction methodology.

**Table 6:** Comparisons of some analytical methods for chromium (III) detection with the proposed method.

Method	Working Range (M)	Detect Limit (M)	pH	Application	Ref.
Extraction-Inductively coupled plasma	0.4 × 10^−9^–1.9 × 10^−6^	0.4 × 10^−9^	6.9–7.8	Lake waters	[6]
Activated alumina/Flame atomic absorption spectrometry	1.9 × 10^−7^– 3.8 × 10^−6^	1.9 × 10^−8^	7.0	Water	[37]
Spectrophotometry	1.9 × 10^−5^– 2.1 × 10^−4^	2.25 × 10^−6^	10	Pharmaceutical sample	[38]
Voltammetry	9.6 × 10^−7^– 1.9 × 10^−5^	1.9 × 10^−9^	4.5	wastewater	[39]
Proposed potentiometry method with SSE	3.0 × 10^−^^9^–1.0 × 10^−^^1^	3.0 × 10^−9^	4–6	Pharmaceutical sample	This work

## 4 Conclusion

In this work, after synthesis and purification of N-(pyridine-2-ylcarbamothioyl) benzamide (NP2YCTB), structural confirmation was done with ^13^C NMR, H NMR and FT-IR spectroscopy. The tendency of NP2YCTB to chromium (III) was confirmed by UV–visible spectrophotometry. It is utilized as an ionophore for creating three different types of PVC membrane electrodes, including solid-state electrodes (SSE) and coated wire electrodes (CWE) as asymmetric electrodes, and liquid membrane electrodes (LME) as symmetric electrodes. 10 % NP2YCTB, 58 % DBP, 2%NaTPB, and 30 % PVC were applied in liquid membrane electrodes which led to a Nernstian slope of 20.7 mV/decade. The membrane's morphology was investigated using FESEM. The findings demonstrate that the membrane's surface is adequately covered with NP2YCTB. Also, the analysis of EDX spectra proved that the membrane matrix included chromium (III) ions. The coordination of NP2YCTB heteroatoms with chromium (III) was confirmed by Fourier transform infrared spectroscopy. The limit of detection for SSE (3 × 10^−9^ mol/L) was enhanced compared with LME (7 × 10^−6^ mol/L) and CWE (3 × 10^−7^ mol/L). The response time of electrodes was very short so it was about 5–6 s for LME and CWE and 5–8 s for SSE. The electrodes had a relatively wide working pH range and high selectivity coefficient values for various cations. The electrodes were successfully utilized to determine the amount of chromium (III) in pharmaceutical tablets and to titrate it with EDTA using potentiometry. All three electrodes showed acceptable performance in chromium (III) measurement, but the solid-state electrode (SSE) was the best electrode because it had a wider working range and lower detection limit.

## Ethical approval

Approved from all the ethical point of view.

## Data availability statement

Data will be made available on request.

No additional information is available for this paper.

## CRediT authorship contribution statement

**Abbas Nasri Fateh:** Investigation, Formal analysis, Data curation. **Leila Hajiaghababaei:** Writing - review & editing, Supervision, Conceptualization. **Mohammad Reza Allahgholi Ghasri:** Supervision, Conceptualization. **Ashraf Sadat Shahvelayati:** Writing - original draft, Conceptualization. **Khadijeh Kalateh:** Methodology, Conceptualization.

## Declaration of competing interest

The authors declare that they have no known competing financial interests or personal relationships that could have appeared to influence the work reported in this paper.
