# Supplementary material for: Fast chromium determination in pharmaceutical tablets by using electrochemical sensors: Preparation and comparison
Source: Heliyon. 2023 Nov 24;9(12):e22842. doi: 10.1016/j.heliyon.2023.e22842 (PMC10731092; doi:10.1016/j.heliyon.2023.e22842)
Supplement: Multimedia component 1 [file mmc1.docx]

**S1**

**Fast chromium determination in pharmaceutical tablets by using electrochemical sensors: Preparation and comparison**

Abbas Nasri Fateh^a^, Leila Hajiaghababaei^*,a^, Mohammad Reza Allahgholi Ghasri^a^ , Ashraf Sadat Shahvelayati^a^, Khadijeh Kalateh^a^

^a^ Department of Chemistry, Yadegar-e-Imam Khomeini (RAH) Shahre Rey Branch, Islamic Azad University, Tehran, Iran.

## **Materials and apparatus**

Sodium tetraphenylborate (NaTPB), higher relative molecular weight poly(vinyl chloride) (PVC), nitrobenzene (NB), dibutyl phthalate (DBP), and tetrahydrofuran (THF) were purchased from Merck Co. and utilized as received. Multiwalled carbon nanotubes were prepared from Sigma-Aldrich and graphite powder with particle size less than 20 µm was bought from Fluka. Epoxy was purchased from Henkel (Germany) and hardener (desmodur RFE) was bought from Bayer (Germany). The utilized nitrate salts of the cations (Merck and Sigma-Aldrich) possessed the maximum available purity. Benzoyl chloride, 2-aminopyridine, potassium thiocyanate, and acetonitrile were purchased from Sigma-Aldrich. Throughout the experiment, double distilled deionized water was utilized. Iodine vapor was utilized for detection in thin layer chromatography (TLC).

## Using a Bruker Tensor 27 tool, Fourier transform infrared spectra were recorded via KBr disks. To record ^13^C and ^1^H nuclear magnetic resonance (NMR) spectra, an Ultra shield Bruker 400 tool was used utilizing CDCl_3_ as the deuterated solvent. A Varian Cary100-Bio UV-visible spectrophotometer was used to record UV-visible spectra. A TESCAN MIRA3 microscope was employed to record Field-emission scanning electron microscopy (FESEM) images. For determining the melting point, a Bransetead Electro Thermal B1 tool was utilized. The potential measurements were conducted at 25.0(±0.1) °C using a multi-meter with a ±0.1 mV precision in voltage measurement (Japan). The Ag/AgCl reference electrodes were internal and external (with Liquid membrane electrode) reference electrodes (Azar-Electrode, Iran).

By determination of the potential difference between the reference electrode and ISE, potentiometric measurements are oriented utilizing a multi-meter at room temperature. The utilized electrochemical cells were as follows:

LME: Ag-AgCl || internal solution, 0.01 mol/L chromium(III) | liquid membrane | chromium test solution || Ag-AgCl, KC1 (satd.)

SSE or CWE: solid-state-membrane or coated wire membrane | chromium test solution || Ag-AgCl, KC1 (satd.)

The solid-state and coated wire electrodes had no internal solution. A small amount of KCl was added to internal solution of LME. To plot the calibration curve, the standard solutions were used and the Debye-Hückel procedure was used to determine the activity values.
